# Burden of High-Risk Human Papillomavirus 16- and 18-Associated Oral Squamous Cell Carcinoma in the Indian Population: A Multiplex Polymerase Chain Reaction Study

**DOI:** 10.7759/cureus.73427

**Published:** 2024-11-11

**Authors:** Nairica E Rebello, Anita E Spadigam, Anita Dhupar

**Affiliations:** 1 Oral and Maxillofacial Pathology, Microbiology and Forensic Odontology, Goa Dental College and Hospital, Panjim, IND

**Keywords:** human papillomavirus, oncogenic viruses, oral squamous cell carcinoma, polymerase chain reaction, virus

## Abstract

Background: Oral squamous cell carcinoma (OSCC) is a multifactorial disease. Despite continuing research, the role and prevalence of human papillomavirus (HPV) in oral carcinogenesis initiation and progression remain elusive.

Aim: This study aimed to detect high-risk HPV 16 and 18 DNA in archival tissue specimens of OSCC using multiplex polymerase chain reaction (PCR) and E6 and E7 DNA in the samples positive for HPV 16 and HPV 18 DNA.

Methods: Detection of viral DNA in 90 samples of OSCC was achieved using multiplex PCR with primers specific for HPV 16 and 18. Positive samples were further subjected to multiplex PCR to detect the presence of viral E6 and E7 DNA.

Results: Among the 90 samples evaluated, 23 (25.6%) were positive for HPV 16 DNA and two (2.2%) for HPV 18 DNA. None showed the presence of both strains in the same sample. Among the 23 samples positive for HPV 16, 17 (73.9%) showed combined expression of E6 and E7 DNA, six (23.1%) expressed E6 DNA alone, and none expressed E7 DNA. Both the samples positive for HPV 18 showed the expression of E7 DNA alone.

Conclusion: The present study establishes the existence of a subset of patients within the Indian subpopulation that harbor the oncogenic strains of HPV. This contributes to the global pool of data and reinforces the need for future research to delve into the role of prophylactic vaccination targeting oncogenic HPV.

## Introduction

Human papillomavirus (HPV) is a unique DNA virus that has been established as the causative agent for a plethora of pathologies, ranging from benign warts to malignancies. It can be categorized as low-, intermediate-, and high-risk based on genotypic variations in the DNA base sequences of E6 and E7 [1]. Genotypes 16 and 18 have been recognized as high-risk and result in dysregulation of cellular mechanisms that control growth and apoptosis by integrating into the host genome [1]. Furthermore, overexpression of viral oncogenes E6 and E7 inactivates tumor suppressor proteins p53 and Rb, respectively, ultimately culminating in uncontrolled cellular proliferation and malignant transformation [1].

The involvement of HPV in oropharyngeal squamous cell carcinoma (OPSCC) is well-established and has been frequently observed worldwide [2]. Features that set apart this distinct entity from HPV-negative OPSCC include better treatment outcomes and prognosis, a predilection for a younger population, and improved response to chemoradiotherapy and immune-checkpoint blockade treatments [3]. Thus, patient stratification based on HPV status would enable the implementation of treatment de-escalation, thereby reducing patient morbidity [3].

The incidence of HPV in oral squamous cell carcinoma (OSCC) in developed countries varies from 10% to 80% [1]. While a significant number of cases of OSCC are attributed to a history of deleterious habits including tobacco and alcohol consumption, there has been a plausible increase in the number of cases with no associated habit history [4]. Data on the role of infectious agents in OSCC in the Indian population is limited due to a majority of cases being attributed to tobacco consumption in various forms [5]. The role of HPV in the context of tobacco-induced carcinogenesis is yet to be established with emphasis on its prognostic value. The present study was undertaken to evaluate the prevalence of high-risk HPV 16 and 18 in OSCC using multiplex polymerase chain reaction (PCR).

## Materials and methods

Sample collection

This study was approved by the Ethics Committee of Goa Dental College and Hospital on January 15, 2018. Since the study is an Institutional Review Board-approved retrospective study on archival tissue samples, patient consent was not applicable. Ninety formalin-fixed paraffin-embedded (FFPE) tissues that were histologically diagnosed as OSCC were retrieved from the departments of Oral and Maxillofacial Pathology and General Pathology of two tertiary care hospitals over a four-year period (2014-2018). Incisional and excisional biopsies were included. Tissues retrieved prior to the year 2014 were excluded due to the risk of inadequate DNA extraction. Incisional biopsies measuring less than 5 mm were excluded due to the insufficient depth of the biopsy. Sections measuring 4 μm were obtained using a manual microtome (Leica Biosystems, Mumbai, India). They were deparaffinized, dehydrated, and stained using hematoxylin and eosin. The stained sections were reported by two observers to confirm the diagnosis of OSCC. These tissues were further processed using multiplex PCR to detect oncogenic HPV 16 and 18. Data on the demographic details of the patient and the clinical course of the disease were obtained from the patient records.

DNA extraction

Sections of 5 microns thick were retrieved from the FFPE tissues. Sections were placed in 1.5 ml Eppendorf tubes and subjected to dehydration. Dehydration was carried out as follows: xylene, 1 ml was added, and the mixture was left undisturbed for 30 minutes. This was followed by centrifugation and elimination of the supernatant. After repeating the previous step, 1 mL of alcohol was added and kept for 30 minutes. This step was repeated, and phosphate-buffered saline (1 ml) was added and kept for 30 minutes. The tubes were centrifuged and the supernatant was discarded. Following dehydration, the pellet was suspended in 500 µl Tris ethylenediamine tetraacetic acid (TE) buffer (1M Tris Buffer, 0.5 ml; 0.5M EDTA, 100 µl; and distilled water, made to 50 ml) and vortexed. The tubes were centrifuged at 5000 revolutions per minute (rpm) for five minutes, and the supernatant was discarded. The step of rinsing with fresh TE buffer was repeated 2-3 times. The supernatant was discarded, 50 µl lysis buffer I (1M TE buffer, 500 µl; Triton X-100, 500 µl; 0.5M EDTA, 100 µl; distilled water, made to 50 ml) was added, the tubes were vortexed, and kept for five minutes. Lysis buffer II (Tris hydrochloric acid (HCl), 50 mM (pH 8.0); potassium chloride (KCl), 50 mM; magnesium chloride (MgCl2), 2.5 mM; Tween 20, 0.45%; Nodient P-40, 0.45%) 50 µl was added along with 10 µl Proteinase-K (10 mg/ml) and vortexed vigorously. The tubes were placed in a water bath at 60˚C for two hours. Enzyme deactivation was achieved by placing the tubes in a boiling water bath for 10 minutes. The supernatant containing DNA was transferred to a clean tube and stored at -20˚C.

Polymerase chain reaction

Multiplex PCR was carried out for the detection of HPV 16 and HPV 18 DNA in one reaction. A total reaction volume of 25 µl was prepared, which contained 1X concentrated Multiplex PCR master mix (Qiagen, Hilden, Germany), 5 pmol of each primer (Bioserve India Pvt Ltd, Hyderabad; Table 1), and 10 pg-1 µg of the template DNA. Qiagen multiplex master mix contains 10X PCR buffer (with 15 mM MgCl_2_); deoxyribonucleotide triphosphate (dNTP) mix, 10 mM of each; and Taq DNA polymerase, 2.5 units/reaction. DNA amplification was performed by placing the PCR tubes in a thermocycler (Applied Biosystems, USA). The thermocycler conditions were 95°C for 15 minutes, followed by 40 cycles of 94°C for 30 seconds; 70°C for 1 minute, 30 seconds, 72°C for 1 minute, extension at 72°C for 5 minutes, and storage at 4°C for 1 minute.

**Table 1:** Primer sequence for human papillomavirus 16 and human papillomavirus 18 DNA

Gene	Primer sequence	Amplified product
HPV-16	Forward 5’- TCCTGCAGGTACCAATGGGGAAGAGG-3’ Reverse 5’-TGCCATACCCGCTGTCTTCGCTTT-3’	397 base pairs
HPV-18	Forward 5’- AACAGTCCATTAGGGGAGCGGCTGGA-3’ Reverse 5’-TGCCGCCATGTTCGCCATTTG-3’	187 base pairs

Agarose gel electrophoresis

The amplified products were separated by 2% agarose gel for the detection of specific bands. The procedure was performed by mixing 2 gm of agarose powder in 100 ml of 1X Tris-acetate-ethylenediaminetetraacetic acid (TAE) buffer (12.1 gm Tris base, 2.850 ml glacial acetic acid, 5 ml 0.5M EDTA, distilled water to adjust volume to 50 ml). The solution was boiled with gentle stirring until a homogenous, clear solution was formed. This was followed by cooling and the addition of 2 µl of ethidium bromide (0.5 µg/ml). The solution was poured into a gel mold, the comb was placed, and it was placed to settle for 20 minutes. Following the removal of the comb, the gel was submerged in 200 ml 1X TAE buffer in the electrophoresis unit (Bio Bee Tech apparatus, Bengaluru, India). Bromophenol blue was added to the amplified samples. The samples were loaded into the wells, and the molecular weight marker (DNA ladder) was loaded in the last well. DNA ladders of 397 base pairs and 187 base pairs were used for HPV 16 and 18, respectively. The gel was run at 60V for two hours. The gel was stained with 0.5 µg/ml ethidium bromide and visualized under a UV transilluminator (Major Science, USA). Bands were recorded using a Gel documentation system (Major Science, USA). The bands were identified, and images were captured and stored.

Subsequently, E6 and E7 DNA were detected in positive samples using gene-specific primers (Table 2) according to the previously described procedure.

**Table 2:** Primer sequence for human papillomavirus 16 and human papillomavirus 18 E6 and E7 DNA

Gene	Primer sequence	Amplified product
HPV-16 E6	Forward 5’-GAAACCGGTTAGTATAAAAGCAGAC-3’ Reverse 5’-AGCTGGGTTTCTCTACGTGTTCT-3’	476 base pairs
HPV-16 E7	Forward 5’-CCATAATATAAGGGGTCGGTGGA-3’ Reverse 5’-TTTTTCCACTAACAGCCTCTACAT-3’	296 base pairs
HPV-18 E6	Forward 5-’AAGATTTATTTGTGGTGT-3’ Reverse 5’-GCTGGATTCAACGGTTTC-3’	477 base pairs
HPV-18 E7	Forward 5’-GGATCCGCATGGACCTAAGGCAACATT-3’ Reverse 5’-GAATTCGCTGCTGGGATGCACACCA-3’	318 base pairs

Statistical analysis

A cross-sectional study design was chosen to evaluate the prevalence of HPV 16 and 18 DNA and E6 and E7 DNA in the study samples.

## Results

**Figure 1:**
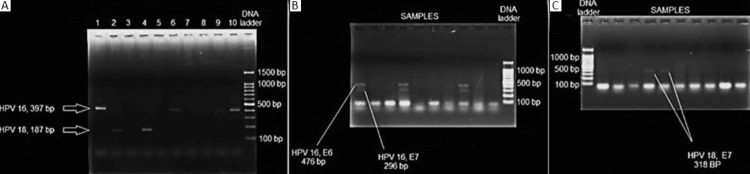
Gel photograph of DNA ladder and samples positive for (A) human papillomavirus 16 DNA (397 base pairs) in lanes 1, 6, 9, and 10 and human papillomavirus 18 DNA (187 base pairs) in lanes 2 and 4; (B) human papillomavirus 16 E6 (476 base pairs) and E7 DNA (297 base pairs) in lanes 1, 4, and 8; and (C) human papillomavirus 18 E7 DNA (318 base pairs) in lanes 5 and 6

Among the 90 samples of OSCC evaluated, HPV-16 DNA was detected in 23 (25.6%) samples (Figure 1A). Human papillomavirus 18 DNA was detected in 2 (2.2%) samples (Figure 1A).

Clinicopathologic parameters of samples positive for HPV 16 and 18 have been presented in Table 3. Among the 23 samples positive for HPV 16 DNA, E6 DNA was detected in six (23.1%) samples, combined expression of E6 and E7 DNA was detected in 17 (73.9%) samples, and E7 DNA was not present in any sample (Figure 1B). E7 DNA was detected in both samples positive for HPV 18 (Figure 1C).

**Table 3:** Clinicopathologic parameters of samples positive for human papillomavirus 16 and 18 HPV: human papillomavirus

Parameter		HPV 16 (n, %)	HPV 18 (n, %)
Sex	Male	13 (56.5%)	1 (50%)
	Female	10 (43.5%)	1 (50%)
Tobacco habit history	Present	16 (69.6%)	0 (0%)
	Absent	7 (30.4%)	2 (100%)
Clinical appearance of the lesion	Proliferative	2 (8.7%)	1 (50%)
	Ulcerative	3 (13%)	0 (0%)
	Ulcero-proliferative	17 (73.9%)	1 (50%)
	Papillary	1 (4.3%)	0 (0%)
Site	Tongue	9 (39.1%)	0 (0%)
	Buccal mucosa	7 (30.4%)	0 (0%)
	Labial mucosa	1 (4.3%)	0 (0%)
	Floor of the mouth	1 (4.3%)	0 (0%)
	Alveolus	5 (21.7%)	1 (50%)
	Lip	0	1 (50%)

## Discussion

Formerly, OSCC was considered a disease primarily caused by tobacco use and alcohol consumption. However, oral cancer secondary to chronic inflammation and infectious agents is currently under investigation [4]. Studies based on the GLOBOCAN 2018 database of cancer incidence have attributed 13% of all cancer diagnoses to infectious agents, a significant yet underestimated value attributed to the lack of detection and documentation, particularly in developing countries [4]. HPV was reported to account for 5900/280000 new cases of OSCC reported in 2018 with a definite male predilection [4]. The present study noted a male preponderance among cases positive for HPV 16 DNA. Yete et al. [5] reviewed 50 studies from across the globe and reported that the average prevalence of HPV-positive oral cancer was 24.4%. In contrast, the prevalence in India was reported at 36.6%, indicating a notably higher rate compared with the global average [5].

The American Society of Clinical Oncology (ASCO) has categorized male gender and tobacco as the main risk factors for oral carcinogenesis [6]. In the present study, a history of tobacco use was observed among most HPV 16 DNA-positive cases.

The eighth edition of the American Joint Committee on Cancer (AJCC) cancer staging manual categorized the staging of OPSCC into two distinct categories: HPV-associated and non-HPV-associated OPSCC [7]. The distinction was based on evidence indicating better prognosis and differing management approaches for the two subtypes. This stratification underscores the importance of screening for HPV before tumor staging and its impact on treatment planning. The same manual also mandates the inclusion of the HPV status of a patient with OSCC in the histopathological report. However, the significance of this parameter in the staging and treatment planning of this subset of patients is yet to be ascertained.

It has been postulated that HPV infection might raise the risk of OSCC by up to three times, regardless of alcohol or tobacco use [8]. Detecting HPV DNA is an initial step in screening for viral infection. However, assessment of the expression of HPV oncogenes E6 and E7 is crucial to confirm the integration of the virus into the host genome and its role in carcinogenesis without other carcinogens [9]. The present study evaluated the expression of E6 and E7 viral DNA in the samples positive for the oncogenic strains of HPV. However, the detection of E6 and E7 RNA was not carried out due to financial constraints. Palve et al. [9] reported a positivity of 15% with HPV E6/E7 RNA in cases positive for HPV 16 and 18 DNA.

A systematic review to evaluate studies reporting the survival of patients with OSCC noted a 53% improved survival rate in HPV-positive OSCC compared with HPV-negative OSCC [10]. Fakhry et al. [11] reported that patients with HPV-positive OSCC demonstrated a better response to chemotherapy and chemoradiotherapy. However, smoking during the course of radiation treatment doubles the risk of mortality [12].

It has been reported that with each additional year of tobacco use, the risk of progression or mortality associated with OSCC increases by 1%, and the risk of developing a second primary tumor rises by 1.5%, even after accounting for HPV tumor status and other relevant factors [12].

Despite the improved survival rates noted in HPV-associated OSCC, it has been reported that there is no difference in quality of life between patients with HPV-positive and HPV-negative oral cancers [12]. This could be attributed to the use of standard treatment modalities in both types of cancer, not accounting for HPV status. The AJCC staging manual mandates staging of oropharyngeal carcinoma based on the presence or absence of HPV to facilitate treatment de-escalation in patients with HPV-associated OPSCC [7]. Thus, further research is imperative to evaluate whether a similar approach to treatment de-escalation would improve the quality of life in patients with HPV-associated OSCC.

The role of vaccination in preventing HPV-associated cervical cancer has been extensively studied. Vaccination of males and females between 9 and 26 years of age has been recommended by the United States Advisory Committee on Immunization Practices (ACEP) [13]. Tsentemeidou et al. [14] conducted a meta-analysis and reported that vaccinated individuals have a 46% lower risk of developing oral HPV infection and an 80% reduced risk of oncogenic HPV 16 infection. Giuliano et al. [15] have currently undertaken a randomized phase III trial to assess the safety and efficacy of the Gardasil 9 vaccine in preventing persistent oral high-risk HPV infections. This could potentially aid in minimizing the risk of OSCC secondary to HPV infection.

Studies indexed in PubMed/Medline reporting the prevalence of HPV in OSCC in the Indian subpopulation have been tabulated in Table 4. The values obtained in the current study are within the range of those obtained in studies conducted within the Indian subcontinent.

**Table 4:** Prevalence of human papillomavirus 16 and 18 in oral squamous cell carcinoma in the Indian subcontinent CISH: chromogen in-situ hybridization; FFPE: formalin-fixed paraffin-embedded; IHC: immunohistochemistry; PCR: polymerase chain reaction

Sample	Sample size	Technique	State/region	Result	Reference
Not stated	106	PCR	Karnataka	43%	Palve et al., 2018 [9]
FFPE	23	PCR	Uttar Pradesh	13%	Dhanapal et al., 2015 [16]
Fresh frozen	30	PCR	Karnataka	60%	Hallikeri et al., 2019 [17]
Not stated	60	PCR	South India	48.3%	Elango et al., 2011 [18]
FFPE	40	PCR	Maharashtra	27.5%	Gheit et al., 2009 [19]
Fresh frozen	86	PCR	North East (Guwahati)	24 cases	Kumar et al., 2015 [20]
Fresh frozen	83	PCR	Mumbai	34%	Koppikar et al., 2006 [21]
FFPE	156	PCR	South India	51.9%	Ramshankar et al., 2014 [22]
Saliva rinse	34	PCR	South India	70.6%	Kulkarni et al., 2011 [23]
Fresh frozen	50	PCR	Rohtak	42%	Parshad et al., 2015 [24]
FFPE	30	IHC	Bengaluru	86.66%	Patil et al., 2014 [25]
Fresh frozen	60	PCR	Karnataka	0%	Rajesh et al., 2017 [26]
FFPE	20	PCR	Andhra Pradesh	55%	Chowdary et al., 2018 [27]
FFPE	31	CISH	Shillong	29%	Jitani et al., 2015 [28]

The current study has some limitations. The definitive causal role of HPV in OSCC in the positive samples could not be established due to an inability to detect E6 and E7 RNA. Additionally, follow-up data was not available for a subset of patients, rendering this crucial parameter unavailable for correlation.

Future scope

The contribution of each carcinogen to the process of carcinogenesis in patients with an HPV infection and tobacco use is yet to be ascertained. It is also essential to determine whether each carcinogen acts independently or if they interact in combination to create a synergistic effect that contributes to carcinogenesis. Considering improved prognosis in patients with HPV-positive OPSCC, an attempt is being made to reduce the toxicity of treatment to improve the quality of life [13]. The application of the same to HPV-positive OSCC and the evaluation of whether it would aid in improving the prognosis of patients with HPV alone versus those with the presence of both HPV and tobacco as carcinogens is yet to be investigated.

## Conclusions

Conclusive evidence on the presence of oncogenic strains of HPV associated with OSCC in the Indian population underscores the need for further studies on specific management protocols tailored for this subset of patients. Additionally, the role of preventive strategies, including vaccination, and its role in reducing disease burden is yet to be established.
